# Absence of a weekday effect on short- and long-term oncologic outcomes of gastrectomy for gastric cancer: a propensity score matching analysis

**DOI:** 10.1186/s12893-022-01756-z

**Published:** 2022-08-06

**Authors:** Tsuneyuki Uchida, Ryuichi Sekine, Kenichi Matsuo, Gaku Kigawa, Takahiro Umemoto, Mikio Makuuchi, Kuniya Tanaka

**Affiliations:** 1 Department of Gastroenterological and General Surgery, Showa University Fujigaoka Hospital, 1-30, Fujigaoka, Aoba-ku, Yokohama, Kanagawa 2278501 Japan; 2 Department of Surgery, Sannodai Hospital, 4-1-38, Higashi-Ishioka, Ishioka, Ibaraki Japan

**Keywords:** Gastric cancer, Gastrectomy, Propensity score matching, Weekday effect, Prognosis

## Abstract

**Background:**

Day of the week when elective gastrointestinal surgery is performed may be influenced by various background and tumor-related factors. Relationships between postoperative outcome and when in the week gastrectomy is performed remain controversial. We undertook this study to evaluate whether weekday of gastrectomy influenced outcomes of gastric cancer treatment (“weekday effect”).

**Methods:**

Patients who underwent curative surgery for gastric cancer between 2004 and 2017 were included in this retrospective study. To obtain 2 cohorts well balanced for variables that might influence clinical outcomes, patients whose gastrectomy was performed early in the week (EW group) were matched 1:1 with others undergoing gastrectomy later in the week (LW group) by use of propensity scores.

**Results:**

Among 554 patients, 216 were selected from each group by propensity score matching. Incidence of postoperative complications classified as Clavien-Dindo grade II or higher was similar between EW and LW groups (20.4% vs. 24.1%; P = 0.418). Five-year overall and recurrence-free survival were 86.0% and 81.9% in the EW group, and 86.2% and 81.1% in the LW group (P = 0.981 and P = 0.835, respectively).

**Conclusions:**

Short- and long-term outcomes were comparable between gastric cancer patients who underwent gastrectomy early and late in the week.

## Background

Gastrectomy is the mainstay of curative treatment for patients with gastric cancer [1]. Combining surgery with chemotherapy and multi-modal treatment has increased survival of patients with resectable gastric cancer [1, 2]. However, overall 5-year postoperative survival is only about 70%, and is strongly dependent on tumor stage at time of surgery: better than 70% for stages I and II, 35% to 54% for stage III, and less than 20% for stage IV [3]. Therefore, we need to identify additional modifiable factors that can improve postoperative prognosis.

Two large studies [4, 5] have associated performance of surgery for gastrointestinal cancer late in the week with greater mortality within 30 postoperative days, attributing this weekday effect to fatigue among surgeons over the course of the week that might impact details of the operation and extent of dissection. These reports also suggested increased risk of tumor recurrence. In contrast, other studies concluded that any weekday effect observed did not alter postoperative prognosis [6, 7], so the relationship between day of surgery and clinical results remains controversial. To our best knowledge, no analyses of this issue in gastric cancer have made adjustments for background factors using propensity score matching.

In the present study we used propensity score matching analysis to minimize distortion from differing patient characteristics with the aim of determining whether late weekday gastrectomy increases postoperative complications or worsens prognosis in patients with gastric cancer.

## Methods

### Study population

Between January 2004 and December 2017, elective gastrectomy to treat primary gastric cancer was performed for 699 consecutive patients at the Department of Gastroenterological and General Surgery of Showa University Fujigaoka Hospital. The study population consisted of single-race patients (Japanese). All elective gastrectomies were performed between Monday and Friday.

**Fig. 1:**
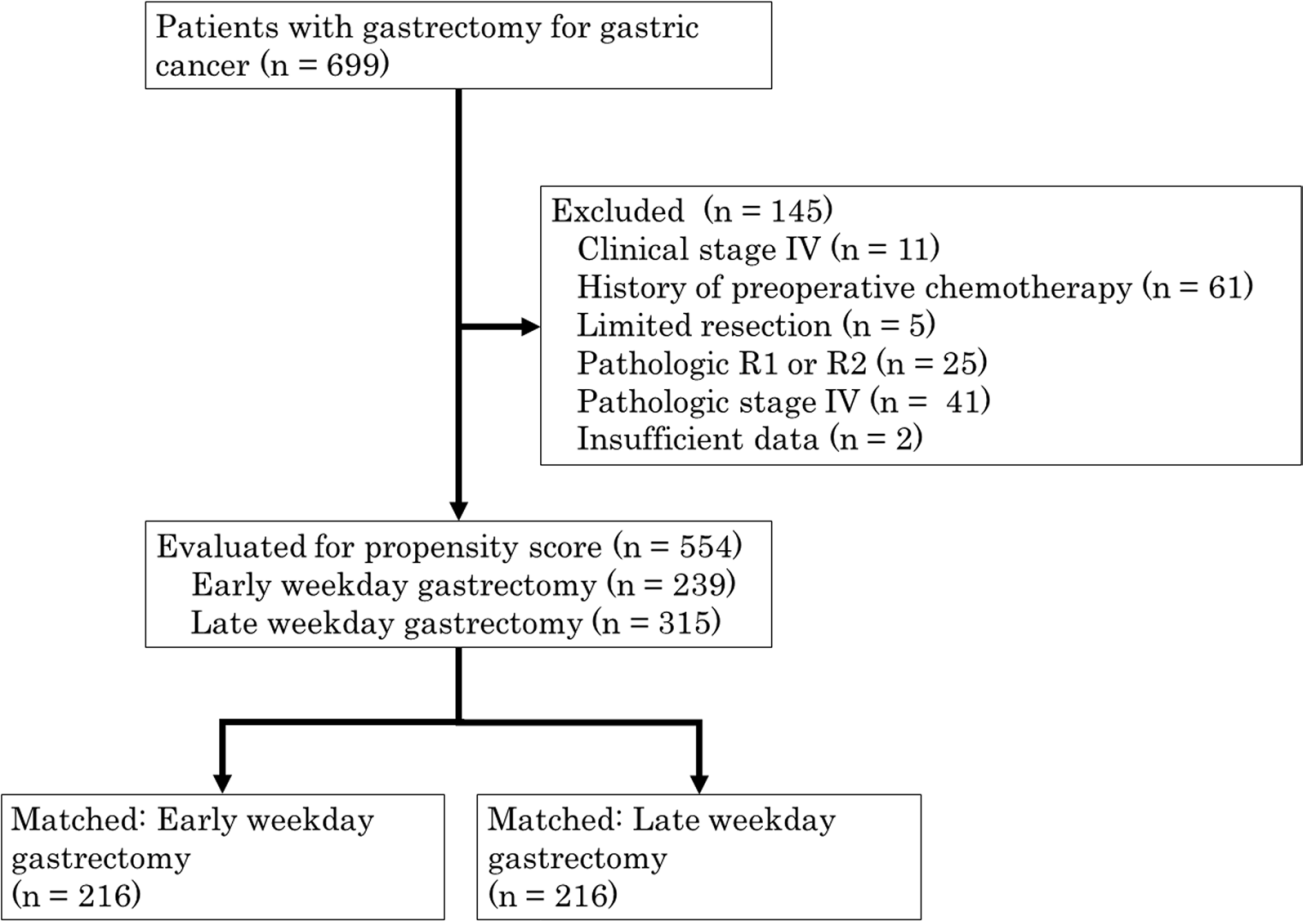
Flow chart of study including patient selection and group assignment

Among these patients, 145 were excluded from the study for the following reasons: preoperative chemotherapy administration in 61 patients; limited gastric resection in 5; palliative gastrectomy defined as R1 or R2 resection [8] in 25; clinical stage IV in 11; pathologic stage IV in 41; and insufficient data for analysis in 2. For the remaining 554 patients, clinical and pathologic data were collected from medical records for retrospective analysis (Fig. 1). Pulmonary function parameters such as vital capacity (VC), %VC, forced expiratory volume (FEV) 1.0, and FEV1.0%, were measured by spirometry. We defined ventilatory impairment as restrictive or obstructive as follows: restrictive ventilatory impairment was %VC below 80% of that predicted, while obstructive ventilatory impairment was FEV1.0% below 70% of forced VC. Data collection and analysis were approved by the institutional review board of Showa University Fujigaoka Hospital (Approval No. F2020C74). All patients provided informed consent for use of anonymous data through an opt-out methodology.

Extent of lymph node dissection was described in accordance with the 2014 Japanese Gastric Cancer Treatment Guidelines (version 4), and cancer staging was based on the 8th edition of the Union for International Cancer Control (UICC) TNM classification system [8, 9].

Analysis was performed for a dichotomized weekday model: Monday and Tuesday were defined as early weekday (EW), while Wednesday through Friday were late weekday (LW), based upon the division of the week used in previous studies of surgical outcome according to when in the week surgery was performed [5, 6]. To confirm similarity of surgical outcomes for the beginning and end of the 5-day work week, a subgroup analysis comparing Monday and Friday surgery was added.

### Postoperative complications

Postoperative complications occurring within 30 days after gastrectomy and assigned a Clavien-Dindo classification of Grade II or higher were reviewed retrospectively [10]. If multiple complications occurred in a single patient, the complication with the highest grade was used for analysis.

### Follow-up and definition of recurrence

Physical examination and blood testing, including tumor markers, were performed every 3 months. Patients underwent at least one type of imaging, usually computed tomography, at 6-month intervals during the first 2 years after surgery and then at 1-year intervals until 5 years after surgery [8]. Recurrence was diagnosed on the basis of findings of radiologic and/or cytologic studies. Even when tumor markers in the blood exceeded normal limits, no diagnosis of recurrence was made before radiologic and/or cytologic evidence had been reviewed. Follow-up data extended to September 2021.

### Propensity score matching

We used propensity score matching to attain optimal balance among baseline variables between EW and LW groups and also between Monday and Friday groups. Propensity scores were estimated using a logistic regression model based on the following 12 variables: age, gender, body mass index (BMI), diabetes mellitus, chronic obstructive pulmonary disease (COPD), serum albumin, carcinoembryonic antigen (CEA), carbohydrate antigen (CA)19-9, impairment of respiratory function, operative procedure, lymph node dissection, and pathologic TNM stage. One-to-one matching without replacement was performed using a 0.2 caliper width, and the resulting score-matched pairs were used in subsequent analyses.

### Statistical analysis

Baseline data are presented as medians with interquartile ranges for continuous variables and as numbers with percentages for categorical variables. The Mann–Whitney *U* test was used to compare continuous variables, while Fisher’s exact test was used to compare categorical variables. Overall survival (OS) was defined as the interval from date of gastrectomy to date of death from any cause. Relapse-free survival (RFS) was defined as the interval from the date of gastrectomy to the date of recurrence of gastric cancer or the date of death from any cause. Surviving patients were censored at the date that they were last known to be alive. Survival was displayed on Kaplan–Meier curves and compared using the log-rank test. Hazard ratios (HR) and 95% confidence intervals (CI) were estimated using the Cox proportional hazards model. *P* values below 0.05 were considered to indicate statistical significance. Statistical analysis was conducted using JMP Pro version 15.0 (SAS Institute, Cary, NC, USA).

## Results

### Patient demographics

In this study 554 patients were enrolled, including 377 men and 177 women with a median age of 70 years. Among all patients, 233 (42.1%) had gastrectomy on Monday, 6 (1.1%) on Tuesday, 110 (19.8%) on Wednesday, 1 (0.2%) on Thursday, and 204 (36.8%) on Friday. Characteristics of the 554 study participants comprising the EW group (n = 239) and the LW group (n = 315) are presented in Table 1. Patients in the LW group had a lower serum albumin concentration (*P* = 0.021) than those in the EW group. After propensity score matching for weekday of gastrectomy, 216 matched pairs were selected (Fig. 1). After matching, characteristics of patients were conserved and no statistically significant differences in characteristics were present between the EW and LW groups (Table 1).

**Table 1:** Comparison of early and late weekday groups (EW and LW) before and after propensity score matching

	All patients (n = 554)		*P* value	Propensity score-matched patients (n = 432)		*P* value
	EW group (n = 239)	LW group (n = 315)		EW group (n = 216)	LW group (n = 216)	
Age, years (median, IQR)	70 (62–77)	70 (63–77)	0.717	70 (62–77)	69 (62–77)	0.889
Gender (n, %)			0.521			0.760
Male	159 (66.5%)	218 (69.2%)		145 (67.1%)	141 (65.3%)	
Female	80 (33.5%)	97 (30.8%)		71 (32.9%)	75 (34.7%)	
Time period (n, %)			0.103			0.101
2004–2010	104 (43.5%)	160 (50.8%)		91 (42.1%)	109 (50.5%)	
2011–2017	135 (56.5%)	155 (49.2%)		125 (57.9%)	107 (49.5%)	
BMI, kg/m^2^ (median, IQR)	22.7 (20.9–24.5)	22.5 (20.3–24.4)	0.161	22.8 (20.9–24.5)	22.7 (20.7–24.9)	0.806
Diabetes mellitus (n, %)			1.000			
Absent	202 (84.5%)	267 (84.8%)		182 (84.3%)	182 (84.3%)	1.000
Present	37 (15.5%)	48 (15.2%)		34 (15.7%)	34 (15.7%)	
COPD (n, %)			0.719			1.000
Absent	226 (94.6%)	295 (93.7%)		203 (94.0%)	202 (93.5%)	
Present	13 (5.4%)	20 (6.3%)		13 (6.0%)	14 (6.5%)	
Hemoglobin, g/dl (median, IQR)	12.7 (11.7–13.9)	12.7 (11.1–13.7)	0.167	12.7 (11.7–13.9)	12.8 (11.6–13.9)	0.803
Albumin, g/dl (median, IQR)	4.1 (3.9–4.4)	4.0 (3.7–4.3)	0.021	4.1 (3.9–4.4)	4.1 (3.8–4.4)	0.750
CEA, ng/ml (median, IQR)	1.6 (0.9–2.6)	1.6 (1.0–2.5)	0.834	1.6 (0.9–2.6)	1.6 (0.9–2.5)	0.983
CA19-9, U/ml (median, IQR)	9.4 (6.0–15.6)	10 (6.8–17.3)	0.161	9.6 (6.4–15.8)	10 (6.5–17.0)	0.371
Impairment of respiratory function (n, %)			0.271			1.000
Absent	156 (65.3%)	220 (69.8%)		141 (65.3%)	140 (64.8%)	
Present	83 (34.7%)	95 (30.2%)		75 (34.7%)	76 (35.2%)	
Clinical tumor depth (n, %)			0.399			0.881
cT1	127 (53.1)	154 (48.9)		116 (53.7%)	121 (56.0%)	
cT2	54 (22.6)	65 (20.6)		49 (22.7%)	47 (21.8%)	
cT3	35 (14.6)	53 (16.8)		34 (15.7%)	29 (13.4%)	
cT4	23 (9.6)	43 (13.7)		17 (7.9%)	19 (8.8%)	
Clinical lymph node metastasis (n, %)			0.056			0.545
cN0	187 (78.2%)	215 (68.3%)		172 (80.0%)	160 (74.1%)	
cN1	40 (16.7%)	72 (22.9%)		34 (15.7%)	44 (20.4%)	
cN2	11 (4.6%)	25 (7.9%)		9 (4.2%)	10 (4.6%)	
cN3	1 (0.4%)	3 (1.0%)		1 (0.5%)	2 (0.9%)	
Clinical TNM stage (n, %)			0.075			0.180
I	165 (69.0%)	193 (61.3%)		150 (69.4%)	149 (69.0%)	
IIA	16 (6.7%)	26 (8.3%)		15 (6.9%)	19 (8.8%)	
IIB	22 (9.2%)	22 (7.0%)		22 (10.2%)	11 (5.1%)	
III	36 (15.1%)	73 (23.2%)		29 (13.4%)	36 (16.7%)	
IVA	0	1 (0.3%)		0	1 (0.5%)	
Operative procedure (n, %)			0.448			0.915
Distal gastrectomy	168 (70.3%)	222 (70.5%)		155 (71.8%)	153 (70.8%)	
Proximal gastrectomy	3 (1.3%)	1 (0.3%)		0	0	
Total gastrectomy	68 (28.4%)	92 (29.2%)		61 (28.2%)	63 (29.2%)	
Operative approach (n, %)			0.059			0.176
Open	107 (44.8%)	167 (53.0%)		91 (42.1%)	106 (49.1%)	
Laparoscopic	132 (55.2%)	148 (47.0%)		125 (57.9%)	110 (50.9%)	
Lymph node dissection (n, %)			0.230			1.000
D1/D1+	127 (53.1%)	150 (47.6%)		117 (54.2%)	116 (53.7%)	
D2 or more	112 (46.9%)	165 (52.4%)		99 (45.8%)	100 (46.3%)	

*BMI* body mass index, *COPD* chronic obstructive pulmonary disease, *IQR* interquartile range, *CEA* carcinoembryonic antigen, *CA* carbohydrate antigen

### Postoperative outcomes and pathologic findings

Among all patients, median operative time and blood loss were 345 min and 265 g, respectively. Operative time and blood loss did not differ between the two groups before or after propensity score matching. Before matching, patients in the LW group had more advanced depth of tumor invasion (*P* = 0.049) and lymph node metastasis (*P* = 0.018) than those in the EW group. After matching, all pathologic variables including depth of tumor invasion ceased to differ significantly among the groups (Table 2).

**Table 2:** Comparison of postoperative outcomes and pathologic findings between early and late weekday groups (EW and LW)

	All patients (n = 554)		*P* value	Propensity score-matched patients (n = 432)		*P* value
	EW group (n = 239)	LW group (n = 315)		EW group (n = 216)	LW group (n = 216)	
Operative time, minutes (median, IQR)	350 (290–421)	345 (277–420)	0.530	350 (290–420)	341 (280–420)	0.703
Operative blood loss, grams (median, IQR)	202 (39–482)	265 (45–645)	0.095	195 (30–460)	230 (40–639)	0.182
Blood transfusion (n, %)			0.581			0.499
Not performed	198 (82.9%)	255 (80.9%)		181 (83.8%)	187 (86.6%)	
Performed	41 (17.1%)	60 (19.1%)		35 (16.2%)	29 (13.5%)	
Hospital stay after surgery, days (median, IQR)	13 (11–17)	13 (11–18)	0.47	13 (10–17)	13 (10–18)	0.907
Death within 30 days after surgery (n, %)	0	2 (0.6%)	0.508	0	2 (0.9%)	0.499
In-hospital death (n, %)	0	4 (1.3%)	0.137	0	2 (0.9%)	0.499
Adjuvant chemotherapy (n, %)			0.211			0.788
Absent	204 (85.4%)	255 (81.0%)		182 (84.3%)	185 (85.7%)	
Present	35 (14.6%)	60 (19.0%)		34 (15.7%)	31 (14.3%)	
Tumor diameter, mm (median, IQR)	35 (25–50)	35 (22–60)	0.318	35 (25–50)	34 (20–50)	0.531
Retrieved number of lymph nodes (median, IQR)	40 (27–50)	38 (26–53)	0.922	39 (27–49)	38 (25–53)	0.930
Pathological tumor depth (n, %)			0.049			0.660
pT1	134 (56.1%)	150 (47.6%)		123 (56.9%)	119 (55.1%)	
pT2	41 (17.1%)	53 (16.8%)		39 (18.1%)	40 (18.5%)	
pT3	43 (18.0%)	61 (19.4%)		39 (18.1%)	35 (16.2%)	
pT4	21 (8.8%)	51 (16.2%)		15 (6.9%)	22 (10.2%)	
Pathological lymph node metastasis (n, %)			0.018			0.844
pN0	173 (72.4%)	204 (64.8%)		156 (72.2%)	161 (74.5%)	
pN1	29 (12.1%)	49 (15.6%)		29 (13.4%)	24 (11.1%)	
pN2	10 (4.2%)	33 (10.5%)		10 (4.6%)	12 (5.6%)	
pN3	27 (11.3%)	29 (9.2%)		21 (9.7%)	19 (8.8%)	
Pathological TNM stage (n, %)			0.075			0.992
IA	120 (50.2%)	136 (43.2%)		109 (50.5%)	109 (50.5%)	
IB	42 (17.6%)	47 (14.9%)		40 (18.5%)	41 (19.0%)	
IIA	24 (10.0%)	35 (11.1%)		23 (10.7%)	24 (11.1%)	
IIB	19 (8.0%)	33 (10.5%)		16 (7.4%)	12 (5.6%)	
IIIA	8 (3.4%)	37 (11.8%)		8 (3.7%)	10 (4.6%)	
IIIB	20 (8.4%)	17 (5.4%)		15 (6.9%)	15 (6.9%)	
IIIC	6 (2.5%)	10 (3.2%)		5 (2.3%)	5 (2.3%)	

*IQR* interquartile range

### Postoperative complications

Details of all complications are shown in Table 3. Considering all 554 patients, morbidity rates was 24.5% (136 patients). Before matching, although the total incidence of complications did not differ significantly among groups (20.5% in the EW group vs. 27.6% in the LW group, *P* = 0.059), the LW group had a greater incidence of pneumonia than the EW group (*P* = 0.006). After matching, total incidence of complications and details of individual complications did not differ significantly between groups.

**Table 3:** Comparison of postoperative complications between early and late weekday groups (EW and LW)

	All patients (n = 554)		*P* value	Propensity score-matched patients (n = 432)		*P* value
	EW group (n = 239)	LW group (n = 315)		EW group (n = 216)	LW group (n = 216)	
All postoperative complications (n, %)	49 (20.5%)	87 (27.6%)	0.059	44 (20.4%)	52 (24.1%)	0.418
Anastomotic leakage	6 (2.5%)	10 (3.2%)	0.800	6 (2.8%)	7 (3.2%)	1.000
Pancreatic fistula	8 (3.4%)	10 (3.2%)	1.000	6 (2.8%)	7 (3.2%)	1.000
Intra-abdominal abscess	6 (2.5%)	14 (4.4%)	0.258	5 (2.3%)	9 (4.2%)	0.416
Anastomotic stenosis	3 (1.3%)	5 (1.6%)	1.000	3 (1.4%)	4 (1.9%)	1.000
Pneumonia	5 (2.1%)	23 (7.3%)	0.006	5 (2.3%)	12 (5.6%)	0.136
Paralytic ileus	5 (2.1%)	2 (0.6%)	0.147	5 (2.3%)	1 (0.5%)	0.216
Stasis syndrome	6 (2.5%)	4 (1.3%)	0.341	5 (2.3%)	2 (0.9%)	0.449
Surgical site infection	2 (0.8%)	3 (1.0%)	1.000	2 (0.9%)	3 (1.4%)	1.000
Urinary-tract infection	2 (0.8%)	3 (1.0%)	1.000	2 (0.9%)	3 (1.4%)	1.000
Bacteremia	2 (0.8%)	8 (2.5%)	0.200	2 (0.9%)	5 (2.3%)	0.449
Delirium	6 (2.5%)	10 (3.2%)	0.800	6 (2.8%)	5 (2.3%)	1.000
Pleural effusion	2 (0.8%)	2 (0.6%)	1.000	2 (0.9%)	1 (0.5%)	1.000
Heart failure	4 (1.7%)	8 (2.5%)	0.567	3 (1.4%)	4 (1.9%)	1.000
Bleeding	2 (0.8%)	0	0.186	2 (0.9%)	0	0.499
Enteritis	1 (0.4%)	3 (1.0%)	0.638	1 (0.5%)	1 (0.5%)	1.000
Other	3 (1.3%)	7 (2.2%)	0.527	3 (1.4%)	3 (1.4%)	1.000

### 30-day mortality and in-hospital deaths

Considering all 554 patients, 30-day mortality and overall mortality rates were 0.4% (2 patients) and 0.7% (4 patients), respectively. One death within 30 days was caused by pneumonia, while the other resulted from invasive infection by *Streptococcus pneumoniae*. One later in-hospital death was caused by heart failure and the other by multiple myeloma. No in-hospital death was a consequence of intra-abdominal infection related or unrelated to anastomotic leakage. No difference between groups was evident in 30-day mortality or other in-hospital deaths between groups (none in the EW group vs. 0.9% in the LW group, *P* = 0.499).

### Survival outcomes

**Fig. 2:**
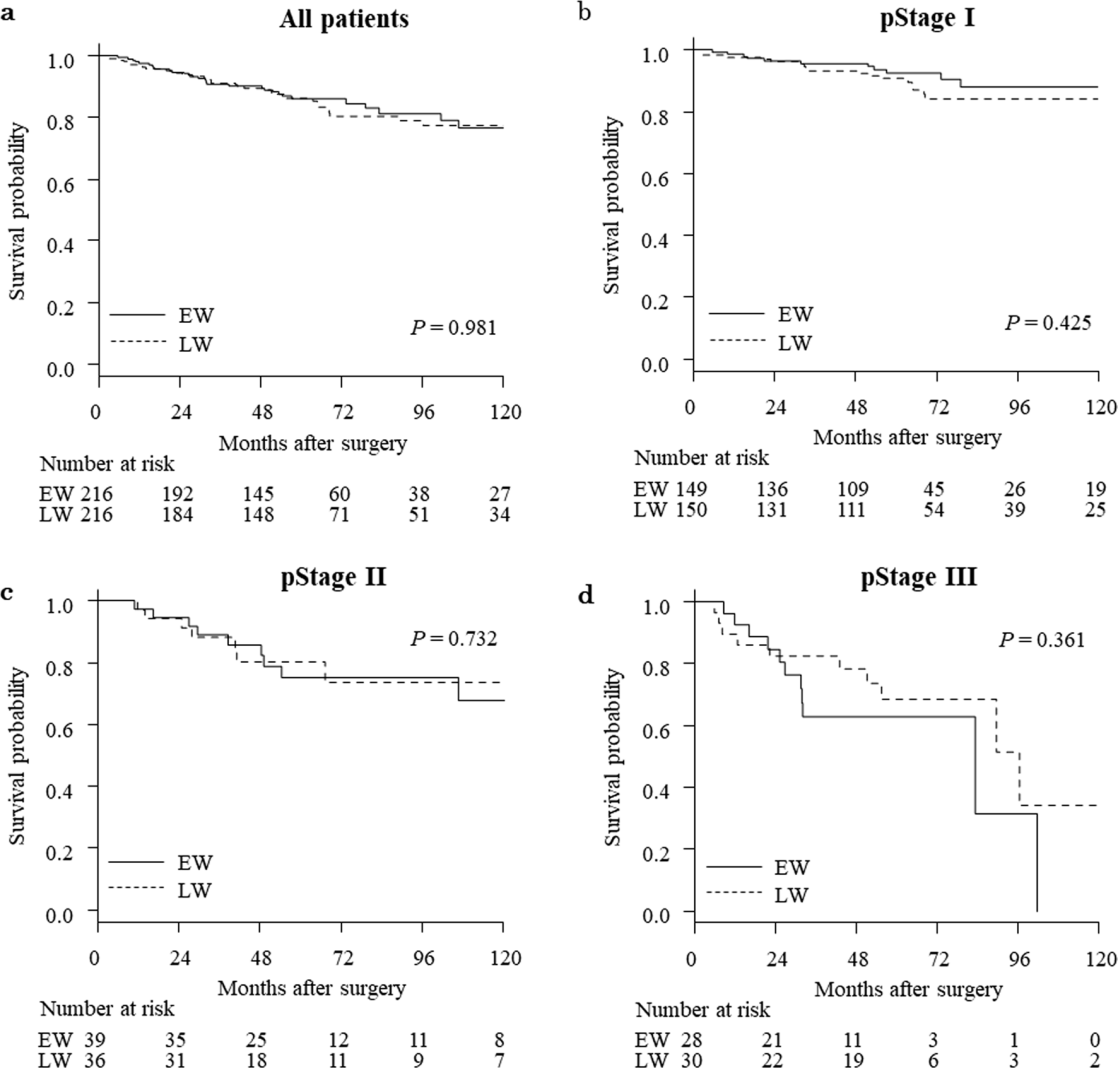
Kaplan–Meier curves comparing EW and LW for overall survival after propensity score matching. **a** All patients, **b** pathologic stage I patients, **c** stage II patients, and **d** stage III patients

**Fig. 3:**
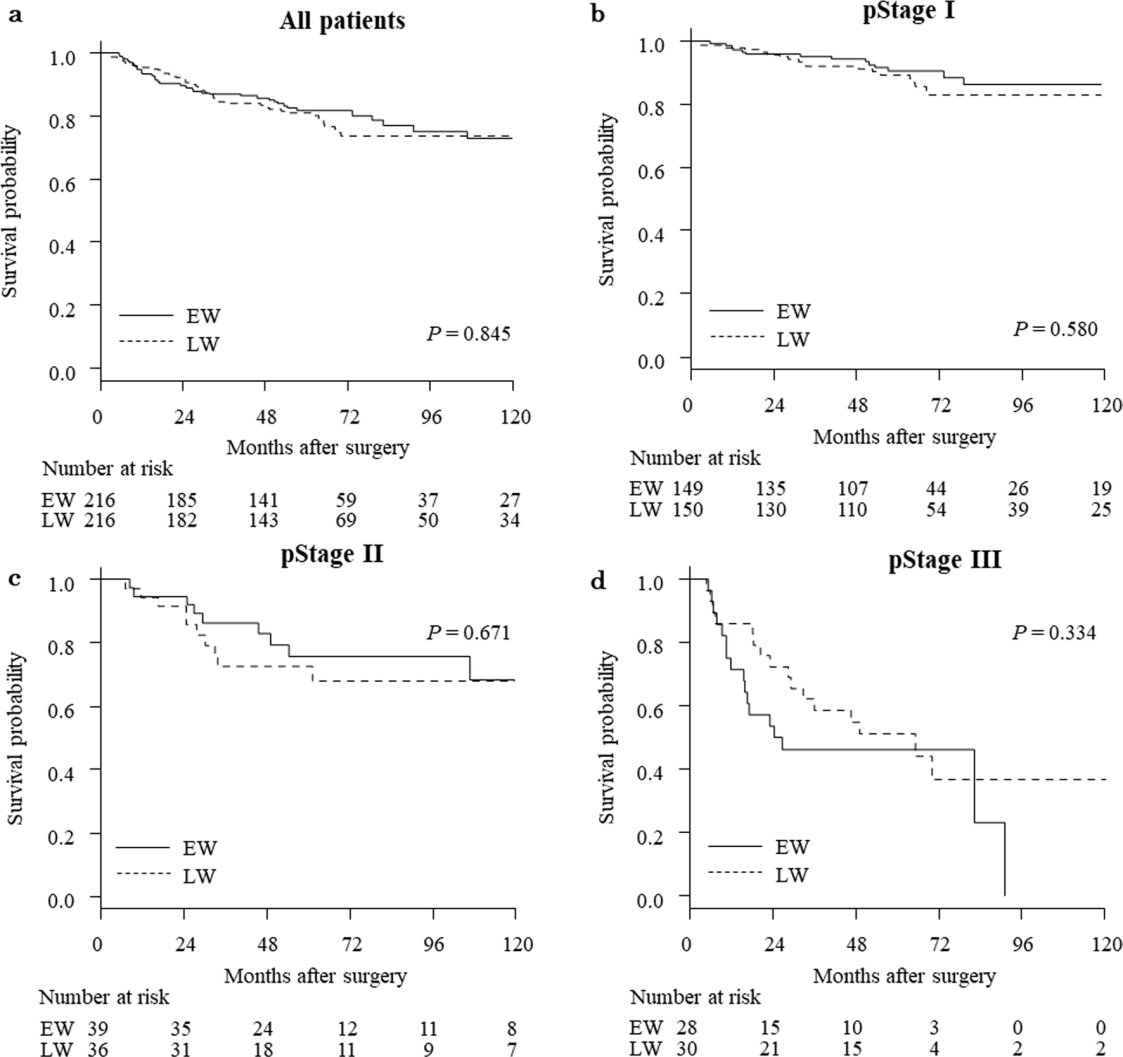
Kaplan–Meier curves comparing EW and LW for recurrence-free survival after propensity score matching. **a** All patients, **b** pathologic stage I patients, **c** stage II patients, and **d** stage III patients

The median observation period was 4.9 years (interquartile range, 3.0 to 6.8 years). Before propensity score matching, 5-year OS rates were 84.8% and 84.0% for the EW and LW groups (*P* = 0.736), while 5-year RFS rates were 80.7% and 78.9% for the EW and LW groups, respectively (*P* = 0.576). Figures 2 and 3 show Kaplan–Meier survival curves for EW and LW groups after propensity score matching. The 5-year OS rate was 86.0% and 86.2% for the EW and LW groups, respectively (*P* = 0.981; Fig. 2A). The HR was 1.01 (95% CI 0.62 to 1.62). Five-year RFS rates were 81.9% and 81.1% for the EW and LW groups, respectively (P = 0.835; Fig. 3A). The HR was 0.96 (95% CI 0.63 to 1.46). When patients were subdivided according to pathologic TNM stage, no significant differences were evident in OS (Fig. 2B–D) or RFS (Fig. 3B–D).

### Subgroup analysis between Monday and Friday surgery

**Fig. 4:**
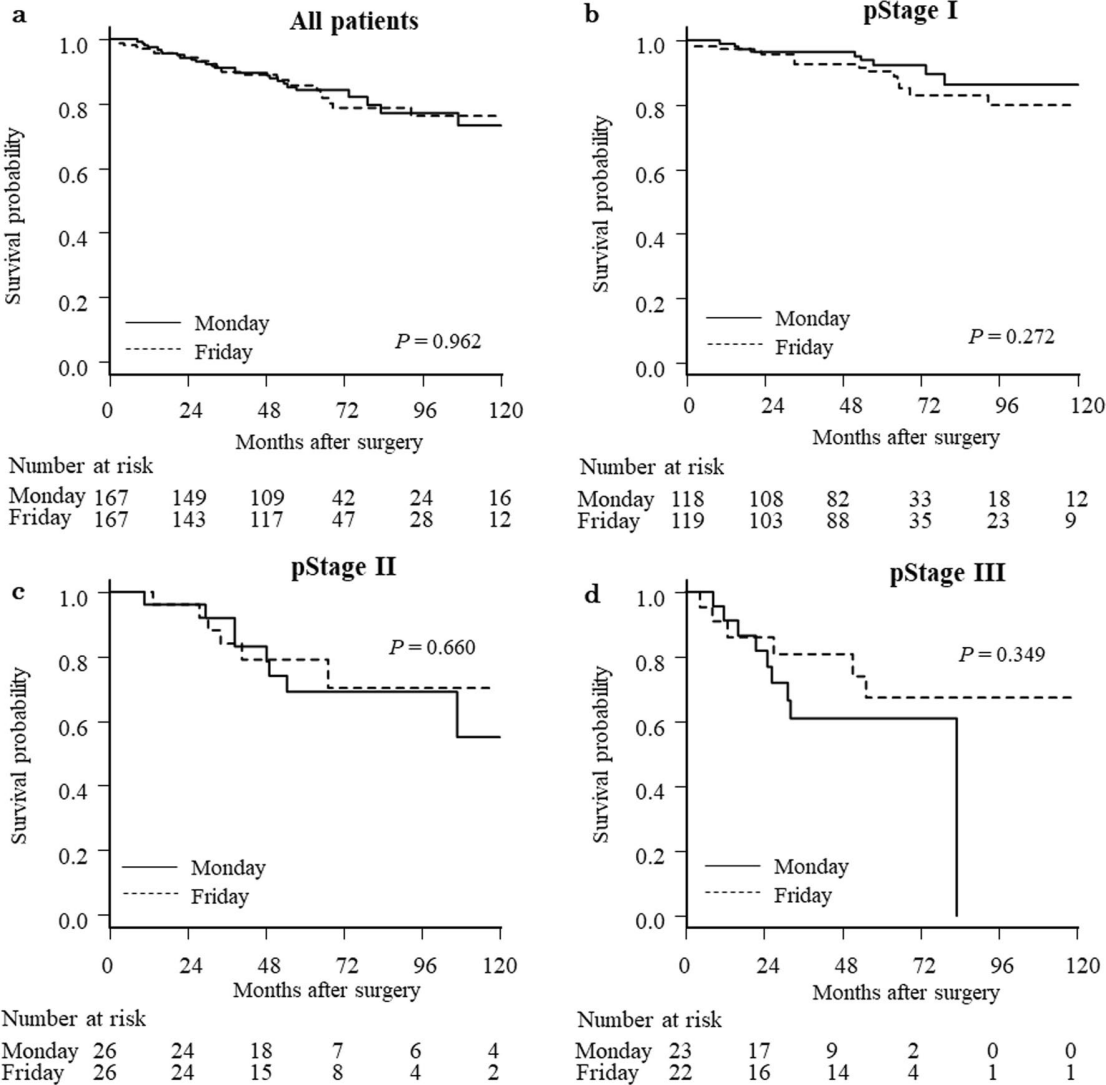
Kaplan–Meier curves comparing Monday and Friday for overall survival after propensity score matching. **a** All patients, **b** pathologic stage I patients, **c** stage II patients, and **d** stage III patients

**Fig. 5:**
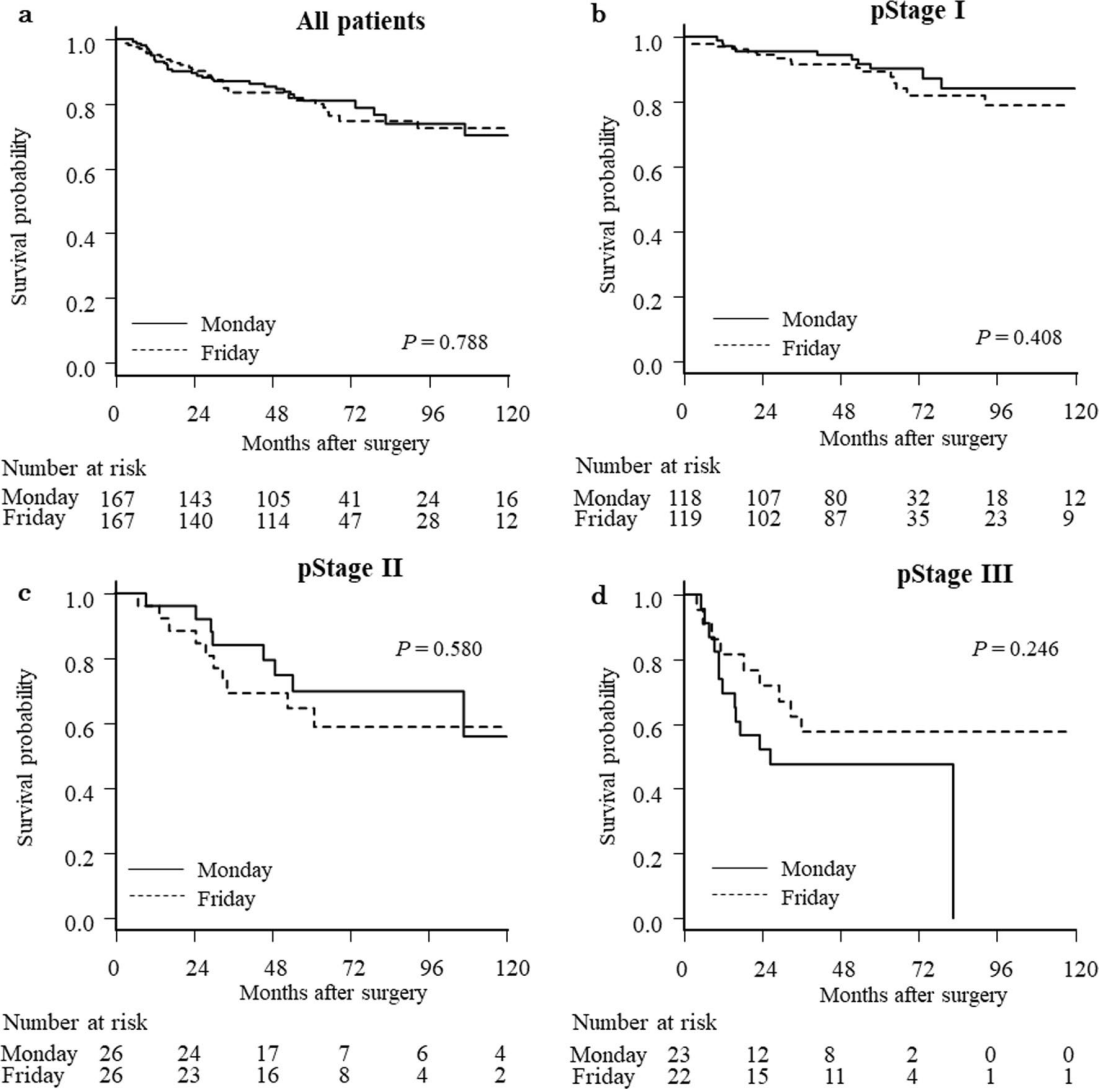
Kaplan–Meier curves comparing Monday and Friday for recurrence-free survival after propensity score matching. **a** All patients, **b** pathologic stage I patients, **c** stage II patients, and **d** stage III patients

Gastric cancer patients underwent surgery on Monday (n = 233) and Friday (n = 204) were included. After propensity score matching, 167 matched pairs were selected. No statistically significant differences in characteristics were present between Monday and Friday groups (Table 4). No statistically significant differences were present between these groups for short-tern postoperative outcomes or pathologic findings (Tables 5 and 6). In addition, no significant differences were evident in OS or RFS between cohorts pathologic TNM stages (Figs. 4 and 5).

**Table 4:** Comparison of Monday and Friday groups before and after propensity score matching

	Patients undergoing surgery on Mon. and Fri. (n = 437)		*P* value	Propensity score-matched patients (n = 334)				*P* value
	Mon. group (n = 233)	Fri. group (n = 204)		Mon. group (n = 167)		Fri. group (n = 167)		
Age, years (median, IQR)	70 (62–77)	69 (62–77)	0.978	70 (62–77)		69 (62–77)		0.855
Gender (n, %)			1.000					1.000
Male	155 (66.5)	135 (66.2)		110 (65.9)		111 (66.5)		
Female	78 (33.5)	69 (33.8)		57 (34.1)		56 (33.5)		
Time period (n, %)			0.244					0.181
2004–2010	104 (44.6)	79 (38.7)		61 (36.5)		74 (44.3)		
2011–2017	129 (55.4)	125 (61.3)		106 (63.5)		93 (55.7)		
BMI, kg/m^2^ (median, IQR)	22.7 (20.9–24.5)	22.5 (20.4–24.8)	0.333	22.6 (21.0–24.5)		22.5 (20.7–25.0)		0.935
Diabetes mellitus (n, %)			0.896					1.000
Absent	197 (84.6)	171 (83.8)		139 (83.2)		140 (83.8)		
Present	36 (15.4)	33 (16.2)		28 (16.8)		27 (16.2)		
COPD (n, %)			1.000					0.620
Absent	220 (94.4)	192 (94.1)		160 (95.8)		157 (94.1)		
Present	13 (5.6)	12 (5.9)		7 (4.2)		10 (6.0)		
Hemoglobin, g/dl (median, IQR)	12.7 (11.7–13.9)	1 2.7 (11.3–13.7)	0.358	12.6 (11.7–13.9)		12.8 (11.6–13.9)		0.848
Albumin, g/dl (median, IQR)	4.1 (3.9–4.4)	4.0 (3.7–4.3)	0.009	4.1 (3.8–4.3)		4.1 (3.8–4.3)		0.799
CEA, ng/ml (median, IQR)	1.5 (0.9–2.5)	1.6 (0.9–2.4)	0.829	1.5 (0.9–2.3)		1.6 (0.9–2.5)		0.920
CA19-9, U/ml (median, IQR)	9.4 (6.0–15.3)	9.4 (6.0–16.6)	0.482	9.4 (6.5–15.0)		9.3 (6.0–16.0)		0.608
Impairment of respiratory function (n, %)			0.543					0.907
Absent	152 (65.2)	139 (68.1)		114 (68.3)		112 (67.1)		
Present	81 (34.8)	65 (31.9)		53 (31.7)		55 (32.9)		
Clinical tumor depth (n, %)			0.763					0.720
cT1	124 (53.2)	115 (56.4)		94 (56.3)		104 (62.3)		
cT2	53 (22.8)	39 (19.1)		36 (21.6)		31 (18.6)		
cT3	33 (14.2)	32 (15.7)		23 (13.8)		21 (12.6)		
cT4	23 (9.9)	18 (8.8)		14 (8.4)		11 (6.6)		
Clinical lymph node metastasis (n, %)			0.672					0.701
cN0	181 (77.7)	215 (74.5)		133 (79.6)		136 (81.4)		
cN1	40 (17.2)	72 (18.6)		24 (14.4)		24 (14.4)		
cN2	11 (4.7)	25 (5.4)		9 (5.4)		5 (3.0)		
cN3	1 (0.4)	3 (1.5)		1 (0.6)		2 (1.2)		
Clinical TNM stage (n, %)			0.486					0.868
I	161 (69.1)	141 (69.1)		120 (71.9)		126 (75.5)		
IIA	16 (6.9)	13 (6.4)		10 (6.0)		9 (5.4)		
IIB	20 (8.6)	11 (5.4)		13 (7.8)		10 (6.0)		
III	36 (15.5)	39 (19.1)		24 (14.4)		22 (13.2)		
Operative procedure (n, %)			0.658					1.000
Distal gastrectomy	163 (70.0)	148 (72.6)		123 (73.7)		122 (73.1)		
Proximal gastrectomy	3 (1.3)	1 (0.5)		0		1 (0.6)		
Total gastrectomy	67 (28.8)	55 (27.0)		44 (26.4)		44 (26.4)		
Operative approach (n, %)			0.099					0.909
Open	107 (45.9)	77 (37.8)		59 (35.3)		61 (36.5)		
Laparoscopic	126 (54.1)	127 (62.3)		108 (64.7)		106 (63.5)		
Lymph node dissection (n, %)			0.924					1.000
D1/D1+	122 (52.4)	108 (52.9)		96 (57.5)		97 (58.1)		
D2 or more	111 (47.6)	96 (47.1)		71 (42.5)		70 (41.9)		

*Mon.* Monday, *Fri.* Friday, *BMI* body mass index, *COPD* chronic obstructive pulmonary disease, *IQR* interquartile range, *CEA* carcinoembryonic antigen, *CA* carbohydrate antigen

**Table 5:** Comparison of postoperative outcomes and pathologic findings between Monday and Friday groups

	Patients undergoing surgery on Mon. and Fri. (n = 437)		*P* value	Propensity score-matched patients (n = 334)		*P* value
	Mon. group (n = 233)	Fri. group (n = 204)		Mon. group (n = 167)	Fri. group (n = 167)	
Operative time, minutes (median, IQR)	350 (290–423)	355 (300–430)	0.458	350 (290–420)	355 (300–430)	0.427
Operative blood loss, grams (median, IQR)	212 (40–487)	100 (20–433)	0.059	103 (20–357)	100 (20–390)	0.911
Blood transfusion (n, %)			1.000			0.199
Not performed	194 (83.3)	170 (83.3)		140 (83.8)	149 (89.2)	
Performed	39 (16.7)	34 (16.7)		27 (16.2)	18 (10.8)	
Hospital stay after surgery, days (median, IQR)	13 (11–18)	12 (10–17)	0.052	13 (10–18)	12 (10–15)	0.054
Death within 30 days after surgery (n, %)	0 (0)	2 (1.0)	0.217	0 (0)	2 (1.2)	0.499
In-hospital death (n, %)	0 (0)	3 (1.5)	0.101	0 (0)	2 (1.2)	0.499
Adjuvant chemotherapy (n, %)			0.438			0.651
Absent	198 (85.0)	167 (81.9)		139 (83.2)	143 (85.6)	
Present	35 (15.0)	37 (18.1)		28 (16.8)	24 (14.4)	
Tumor diameter, mm (median, IQR)	35 (25–50)	35 (20–60)	0.450	31 (22–50)	32 (20–51)	0.992
Retrieved number of lymph nodes (median, IQR)	40 (27–50)	39 (28–54)	0.514	40 (29–49)	37 (26–53)	0.986
Pathological tumor depth (n, %)			0.207			0.839
pT1	130 (55.8)	110 (53.9)		102 (61.1)	99 (59.3)	
pT2	41 (17.6)	28 (13.7)		26 (15.6)	25 (15.0)	
pT3	41 (17.6)	35 (17.2)		25 (15.0)	24 (14.4)	
pT4	21 (9.0)	31 (15.2)		14 (8.4)	19 (11.4)	
Pathological lymph node metastasis (n, %)			0.634			0.520
pN0	170 (73.0)	144 (70.6)		122 (73.1)	130 (77.8)	
pN1	26 (11.2)	27 (13.2)		20 (12.0)	18 (10.8)	
pN2	10 (4.3)	13 (6.4)		9 (5.4)	4 (2.4)	
pN3	27 (11.6)	20 (9.8)		16 (9.6)	15 (9.0)	
Pathological TNM stage (n, %)			0.118			0.999
IA	117 (50.2)	104 (51.0)		92 (55.1)	93 (55.7)	
IB	41 (17.6)	26 (12.8)		26 (15.6)	26 (15.6)	
IIA	24 (10.3)	17 (8.3)		14 (8.4)	16 (9..6)	
IIB	17 (7.3)	20 (9.8)		12 (7.2)	10 (6.0)	
IIIA	8 (3.4)	19 (9.3)		8 (4.8)	8 (4.8)	
IIIB	20 (8.6)	12 (5.9)		12 (7.2)	11 (6.6)	
IIIC	6 (2.6)	6 (2.9)		3 (1.8)	3 (1.8)	

*Mon.* Monday, *Fri.* Friday, *IQR* interquartile range

**Table 6:** Comparison of postoperative complications between Monday and Friday groups

	Patients undergoing surgery on Mon. and Fri. (n = 437)		*P* value	Propensity score-matched patients (n = 334)		*P* value
	Mon. group (n = 233)	Fri. group (n = 204)		Mon. group (n = 167)	Fri. group (n = 167)	
All postoperative complications: n (%)	47 (20.2)	53 (26.0)	0.171	35 (21.0)	39 (23.4)	0.693
Anastomotic leakage	5 (2.2)	6 (2.9)	0.762	5 (3.0)	5 (3.0)	1.000
Pancreatic fistula	8 (3.4)	5 (2.5)	0.587	6 (3.6)	4 (2.4)	0.750
Intra-abdominal abscess	6 (2.6)	6 (2.9)	1.000	5 (3.0)	4 (2.4)	1.000
Anastomotic stenosis	3 (1.3)	5 (2.5)	0.482	3 (1.8)	4 (2.4)	1.000
Pneumonia	5 (2.2)	15 (7.4)	0.011	3 (1.8)	9 (5.4)	0.139
Paralytic ileus	5 (2.2)	2 (1.0)	0.457	5 (3.0)	1 (0.6)	0.215
Stasis syndrome	6 (2.6)	1 (0.5)	0.128	3 (1.8)	0	0.248
Surgical site infection	2 (0.9)	3 (1.5)	0.668	1 (0.6)	3 (1.8)	0.623
Urinary-tract infection	2 (0.9)	2 (1.0)	1.000	2 (1.2)	2 (1.2)	1.000
Bacteremia	2 (0.9)	3 (1.5)	0.668	1 (0.6)	3 (1.8)	0.623
Delirium	6 (2.6)	9 (4.4)	0.307	5 (3.0)	4 (2.4)	1.000
Pleural effusion	2 (0.9)	1 (0.5)	1.000	2 (1.2)	1 (0.6)	1.000
Heart failure	4 (1.7)	3 (1.5)	1.000	2 (1.2)	2 (1.2)	1.000
Bleeding	2 (0.9)	0	0.501	2 (1.2)	0	0.499
Enteritis	0	0		0	0	
Other	3 (1.3)	3 (1.5)	1.000	2 (1.2)	2 (1.2)	1.000

*Mon.* Monday, *Fri.* Friday

## Discussion

Our results indicated absence of significant differences in postoperative complications and 30-day mortality between EW and LW groups. The LW group experienced OS and RFS similar to those for the EW group. A subgroup analysis comparing Monday with Friday surgery also showed similar results. To the best of our knowledge, the impact of operative timing within the week for gastrectomy to treat gastric cancer on short- and long-term outcomes according to propensity score matching analysis has not been reported previously.

In this study, patients in the LW group had significantly lower serum albumin concentrations, greater tumor depth, and more advanced lymph node metastases. Similarly to our results, other studies have reported larger numbers of advanced cancer cases undergoing surgery later in the week [5, 11], but the reason for this is not clear. No significant difference was evident between our two groups concerning postoperative complications in general or 30-day mortality. However, our LW group showed a significantly higher incidence of postoperative pneumonia than EW group patients. Advanced gastric cancer and poor nutrition may have contributed to the development of pneumonia [12, 13]. After propensity score matching, no significant differences were evident between EW and LW groups either for postoperative complications including pneumonia or for 30-day mortality. Previous studies found that operative timing during the week when esophagectomy or gastrectomy was performed did not significantly influence risk of 30- or 90-day mortality, but details of postoperative complications were not given [6, 14, 15]. After we adjusted for variables including hypoalbuminemia and pathologic TNM stage, we found no significant difference between groups in incidence of any individual postoperative complication or in 30-day mortality.

Previous studies have examined effects of day of the week when cancer surgery was performed on long-term outcomes [4, 16]. As for gastric cancer surgery, a recent study from the Netherlands found gastrectomy performed late in the week to be associated with a lower lymph node yield than gastrectomy early in the week [15]. However, conclusive evidence has not been found for significant impact of day of surgery on postoperative survival in patients undergoing gastrectomy for gastric cancer [14, 15]. In some reports nutritional status, surgical procedures, extent of lymph node dissection, postoperative complications, and pathologic stage, which often are associated with postoperative prognosis, were not described in detail. In our study these background factors were investigated thoroughly. Patients in the LW group had significantly lower serum albumin, greater tumor depth, and more advanced lymph node metastases than those in EW group. We therefore minimized effect of such background factors using propensity score matching between EW and the LW groups, after which we found no difference between groups, not only for number of lymph nodes dissected but also for long-term prognosis at any stage. A previous report used propensity score matching analysis to isolate the influence of day of surgery on subsequent mortality, finding excess mortality following Friday procedures [17]; however, that study concerned elective colorectal resections for inflammatory bowel disease and other non-oncologic indications as well as malignant disease. Our study is the first to demonstrate that after adjustment for confounding variables, long-term gastric cancer outcomes for patients at any disease stage after gastrectomy were not influenced by weekday of surgery.

Our results demonstrated absence of a need to restrict surgery for gastric cancer to early weekdays. A surgeon may be better rested early in the work week than later because sleep deficits and fatigue could accrue as the week progresses. The contribution of physician fatigue to human error has become a major concern, and has led to enactment of work-hours limitations [18]. Studies using surgical simulation tools have associated sleep deprivation with increased technical errors [19, 20]. Further, previous studies found that a variety of surgical procedures performed on Friday were associated with higher 30-day mortality than similar surgery early in the week [21, 22]. However, our propensity-matched study demonstrated that early vs. late weekday surgery was not associated with differences in postoperative outcome after gastrectomy for gastric cancer. Two factors might explain this lack of a weekday effect. First, surgical indications and preoperative, operative, and postoperative procedures for gastric cancer patients are defined by standardized guidelines in Japan [8], which could help to maintain quality of perioperative management even when the surgeon is tired or the number of staff is reduced. Perioperative management and treatment for postoperative complications are less likely to be dependent on the day of the week. According to a nationwide Japanese database, 30-day mortality is low for both distal gastrectomy (0.6%) and total gastrectomy (1.0%) [23], and our findings are consistent with the results of that report. Second, surgeons in this study may have been similarly rested early and late in the week. At our hospital, we perform emergency surgery for acute abdomen and similar clinical situations as well as elective surgery for malignant disease throughout the week. Effects involving attending surgeons’ sleep time during the week may be lessened by surgeons taking turns during case assignment. Further investigation of the influence of sleep time on surgical care is warranted.

Some limitations are evident in our present study. First, its retrospective nature and single-institutional setting may have biased the data. Second, our study excluded patients who received neoadjuvant chemotherapy. While neoadjuvant chemotherapy is considered standard treatment for gastric cancer patients in most Western countries according to several reports [24, 25], it is not recommended as the standard treatment under Japanese guidelines since its effectiveness remains to be fully proven in the Japanese population [8]. Furthermore, the drugs given and duration of chemotherapy have changed during the time interval applicable to patients considered for this study. Therefore, patients who received neoadjuvant chemotherapy were excluded. Third, the number of gastrectomies on Tuesdays and Thursdays was extremely small because of a need to coordinate operating room schedules with other surgical services. We conducted a subgroup comparison between Monday and Friday surgery to supplement the comparison between EW and LW groups. Here too, the subgroups showed absence of a weekday effect on short- and long-term oncologic outcomes of gastrectomy for gastric cancer. Fourth, although many background factors that might have affected short- and long-term postoperative outcomes were adjusted for by our propensity score matching process, unknown variables that we failed to consider as covariates may have affected our analysis.

## Conclusion

The present study demonstrated that short- and long-term postoperative results for gastric cancer were not affected by the weekday when gastrectomy was performed, and operative scheduling for gastric cancer patients need not be limited to early weekdays.

## Data Availability

The datasets used and/or analyzed during the current study are available from the corresponding author on reasonable request.

## References

[1] Sakuramoto S, Sasako M, Yamaguchi T, Kinoshita T, Fujii M, Nashimoto A (2007). Adjuvant chemotherapy for gastric cancer with S-1, an oral fluoropyrimidine. N Engl J Med.

[2] Bang YJ, Kim YW, Yang HK, Chung HC, Park YK, Lee KH (2012). Adjuvant capecitabine and oxaliplatin for gastric cancer after D2 gastrectomy (CLASSIC): a phase 3 open-label, randomised controlled trial. Lancet.

[3] Katai H, Ishikawa T, Akazawa K, Isobe Y, Miyashiro I, Oda I (2018). Five-year survival analysis of surgically resected gastric cancer cases in Japan: a retrospective analysis of more than 100,000 patients from the nationwide registry of the Japanese Gastric Cancer Association (2001–2007). Gastric Cancer.

[4] Lagergren J, Mattsson F, Lagergren P (2017). Weekday of cancer surgery in relation to prognosis. Br J Surg.

[5] Lagergren J, Mattsson F, Lagergren P (2016). Weekday of esophageal cancer surgery and its relation to prognosis. Ann Surg.

[6] Visser E, van Rossum PSN, Verhoeven RHA, Ruurda JP, van Hillegersberg R (2017). Impact of weekday of esophagectomy on short-term and long-term oncological outcomes: a nationwide population-based cohort study in the Netherlands. Ann Surg.

[7] Frostberg E, Christensen RD, Rahr HB (2019). The day of week of elective colorectal cancer surgery has no impact on mortality and morbidity. Dan Med J.

[8] Japanese Gastric Cancer Association (2017). Japanese gastric cancer treatment guidelines 2014 (ver. 4). Gastric Cancer.

[9] Brierley JD, Gospodarowicz MK, Wittekind C (2017). Gastrointestinal tumours. TNM classification of malignant tumours.

[10] Dindo D, Demartines N, Clavien PA (2004). Classification of surgical complications: a new proposal with evaluation in a cohort of 6336 patients and results of a survey. Ann Surg.

[11] Voeten DM, Elfrink AKE, Gisbertz SS, Ruurda JP, van Hillegersberg R, van Berge Henegouwen MI (2021). Minimally invasive oncologic upper gastrointestinal surgery can be performed safely on all weekdays: a nationwide cohort study. World J Surg.

[12] Kiuchi J, Komatsu S, Ichikawa D, Kosuga T, Okamoto K, Konishi H (2016). Putative risk factors for postoperative pneumonia which affects poor prognosis in patients with gastric cancer. Int J Clin Oncol.

[13] Baba H, Tokai R, Hirano K, Watanabe T, Shibuya K, Hashimoto I (2020). Risk factors for postoperative pneumonia after general and digestive surgery: a retrospective single-center study. Surg Today.

[14] Berlth F, Messerle K, Plum PS, Chon SH, von Ambüren J, Hohn A (2018). Impact of the weekday of surgery on outcome in gastric cancer patients who underwent D2-gastrectomy. World J Surg.

[15] Visser E, Brenkman HJF, Verhoeven RHA, Ruurda JP, van Hillegersberg R (2017). Weekday of gastrectomy for cancer in relation to mortality and oncological outcomes—a Dutch population-based cohort study. Eur J Surg Oncol.

[16] Njølstad TS, Werner HM, Marcickiewicz J, Tingulstad S, Staff AC, Oddenes K (2017). Late-week surgical treatment of endometrial cancer is associated with worse long-term outcome: Results from a prospective, multicenter study. PLoS ONE.

[17] Vohra RS, Pinkney T, Evison F, Begaj I, Ray D, Alderson D (2015). Influence of day of surgery on mortality following elective colorectal resections. Br J Surg.

[18] Kelz RR, Freeman KM, Hosokawa PW, Asch DA, Spitz FR, Moskowitz M (2008). Time of day is associated with postoperative morbidity an analysis of the national surgical quality improvement program data. Ann Surg.

[19] Eastridge BJ, Hamilton EC, O'Keefe GE, Rege RV, Valentine RJ, Jones DJ (2003). Effect of sleep deprivation on the performance of simulated laparoscopic surgical skill. Am J Surg.

[20] Whelehan DF, McCarrick CA, Ridgway PF (2020). A systematic review of sleep deprivation and technical skill in surgery. Surgeon.

[21] Aylin P, Alexandrescu R, Jen MH, Mayer EK, Bottle A (2013). Day of week of procedure and 30 day mortality for elective surgery: retrospective analysis of hospital episode statistics. BMJ.

[22] Zare MM, Itani KM, Schifftner TL, Henderson WG, Khuri SF (2007). Mortality after nonemergent major surgery performed on Friday versus Monday through Wednesday. Ann Surg.

[23] Hasegawa H, Takahashi A, Kakeji Y, Ueno H, Eguchi S, Endo I (2019). Surgical outcomes of gastroenterological surgery in Japan: report of the National Clinical Database 2011–2017. Ann Gastroenterol Surg.

[24] Cunningham D, Allum WH, Stenning SP, Thompson JN, Van de Velde CJ, Nicolson M (2006). Perioperative chemotherapy versus surgery alone for resectable gastroesophageal cancer. N Engl J Med.

[25] Al-Batran SE, Homann N, Pauligk C, Goetze TO, Meiler J, Kasper S (2019). Perioperative chemotherapy with fluorouracil plus leucovorin, oxaliplatin, and docetaxel versus fluorouracil or capecitabine plus cisplatin and epirubicin for locally advanced, resectable gastric or gastro-oesophageal junction adenocarcinoma (FLOT4): a randomised, phase 2/3 trial. Lancet.

